# Social media and body dissatisfaction in young adults: An experimental investigation of the effects of different image content and influencing constructs

**DOI:** 10.3389/fpsyg.2023.1037932

**Published:** 2023-03-08

**Authors:** Raquel Castellanos Silva, Gisela Steins

**Affiliations:** Faculty of Educational Sciences, Institute of Psychology, University of Duisburg-Essen, Essen, Germany

**Keywords:** body dissatisfaction, social media, beauty ideals, body diversity, online survey, moderation, mediation, group differences

## Abstract

Research shows negative correlations between media exposure of body images in the context of hegemonic beauty ideals and body satisfaction. The present study deals with the underlying mechanisms and the effects of different exposure contents. In the online experimental study, a sample consisting of 226 individuals (82.3% female, 17.7% male) received a three-minute exposure to Instagram images of women and men in the context of either hegemonic beauty ideals in the experimental group or body diversity in the control group. A conducted Mixed ANOVA with repeated measures showed significant group differences, including an increase in body dissatisfaction in the experimental group and a reduction in the control group after exposure. Statistically significant detrimental effects of exposure to images in the experimental group on women’s state mood as well as descriptive similar tendencies on men’s state mood were found. Moderating effects of the tendency to make upward social comparisons and the internalization of the gender-specific beauty ideal on the relationship between exposure content and the change scores of body dissatisfaction were found. Furthermore, a mediation model was calculated to investigate the effect of exposure content on post-measurement of body dissatisfaction, using the constructs “comparison processes regarding sexual attractiveness” and “assessment of one’s own sexual attractiveness” as mediators. The model did not yield significant mediation, although significant relationships were found between the model components. Exploratory analyses were conducted on the influence of the assessment of one’s own sexual attractiveness on related social comparisons and the intensity of engagement with Instagram content as a predictor of body dissatisfaction. The results highlight the relevance for psychoeducational purposes of addressing a critical engagement with depicted beauty ideals in social media. Moreover, the study proposes body diversity as an alternative content that can have a positive impact on body satisfaction, which can be actively sought during the individual Instagram user experience.

## Introduction

Body dissatisfaction is a widespread phenomenon that encompasses an individual’s negative thoughts and feelings about their own body and appearance. Body dissatisfaction includes, on the one hand, the subjective evaluation of appearance and, on the other hand, the perceived discrepancy between actual and desired states related to physical appearance (Grogan, 1999; Blake, 2015). This is not a stable individual characteristic. Rather, the attitude towards one’s own appearance is cognitively and affectively based and can thus vary under the influence of various personality-psychological or sociocultural factors (Blake, 2015). The phenomenon is observable in both women and men (Fiske et al., 2014) and can occur across the lifespan (Esnaola et al., 2010; Bucchianeri et al., 2013; Tatangelo et al., 2016). The characteristics attributed the greatest relevance to body dissatisfaction change in older adults. Concerns about variables such as body shape or weight decrease with age (Vega et al., 2015; Cameron et al., 2019). In a 10-year longitudinal study, Bucchianeri et al. (2013) found that, in a north-American sample, body dissatisfaction increased during the transition of middle school to high school in both sexes, and increased even further during the period from high school to young adulthood, in association with an increase in BMI. Nonetheless, even when controlling for BMI, both sexes reported high levels of bodily dissatisfaction during young adulthood and no reduction of body dissatisfaction was found during this period, which highlights the relevance of research in this age group.

Body dissatisfaction is associated with limitations in psychological well-being and perceived quality of life (McLean and Paxton, 2019). In terms of psychopathologies, body dissatisfaction is thought to play an important etiological role in eating disorders (Margraf and Schneider, 2018), including an upholding role in the symptomatology binge-eating disorder (Srivastava et al., 2020). Moreover, body dissatisfaction is one of the main risk factors that predicted the development of bulimia nervosa in multiple studies, and has been theorized to be a risk factor for anorexia nervosa (Stice and Shaw, 2002; Stice, 2016). In a review of prospective studies, Stice (2016) found that body dissatisfaction was the most consistently identified risk factor predicting any eating disorder among several studies. The results of some other studies point not only to an association between engagement with social media content and an increased risk of anorexia nervosa, but also an increased risk of an eating disorder in the first place, e.g., López-Guimera et al. (2010) or Lee-Won et al. (2020). Furthermore, body dissatisfaction is related to psychopathologies such as depression (Noles et al., 1985; Brechan and Kvalem, 2015), anxiety disorders (Fitzsimmons-Craft and Bardone-Cone, 2012) or body dysmorphic disorder (Martin and Buhlmann, 2020). At a subclinical level, the presence of body dissatisfaction is also associated with sometimes harmful appearance modification behaviors, such as aesthetic surgery, excessive exercise, or use of laxatives or steroids (Brennan et al., 2010). Other associations are with potentially harmful health behaviors that are not associated with a desire for body modification but may result from body dissatisfaction, such as lower willingness to self-examine for cancer prevention (Ridolfi and Crowther, 2013), higher rates of smoking (Potter et al., 2004), reduced quality of life (Griffiths et al., 2016), and sexual dysfunction (Davidson and McCabe, 2005).

Cultural socialization can account for the association between exposure to beauty ideals and body dissatisfaction through several mechanisms. One of the best known models in body dissatisfaction research is the Tripartite Influence Model by Thompson et al. (1999). According to this model, the internalization of media beauty ideals and the tendency to make appearance-related comparisons influence the relationship between social influence and body dissatisfaction. The social influence comes from three sources: the parents, the peer group and the media. Body dissatisfaction, in turn, favors disturbed eating behavior in women, often with the goal of weight reduction, and muscle-building behavior in men (Tylka, 2011). Within the framework of Festinger (1954) theory of social comparisons, two possible directions of comparison are postulated, on the one hand upward, with accompanying negative effects on self-worth, or downward, with a positive effect on self-worth. Sources of social comparison are people who enable a realistic comparison, e.g., in the case of a social comparison regarding appearance, a comparison group could be people of the same sex at approximately the same age. Recent research found that the tendency to make social comparisons of appearance may have a mediating role in the effect of exposure to idealized body images on body dissatisfaction (Tiggemann and Zaccardo, 2015; Brown and Tiggemann, 2016). Accordingly, based on this theory, the comparison processes with one’s own gender based on the experienced similarity are recorded in particular. In addition, however, the idea of what the opposite sex (or the sexually preferred sex) finds sexually attractive is an important factor with regard to the effects of information about one’s own appearance derived from social comparisons (Bergstrom et al., 2004), so that comparisons with regard to sexual attractiveness can be of relevance for body dissatisfaction. The criteria for assessing one’s sexual attractiveness are the same as observers would use (Wade, 2000), which is consistent with Fredrickson and Roberts (1997) Objectification Theory, which states that individuals tend to view their appearance from an observer’s perspective. Fioravanti et al. (2021b) found that womens’ assessment of their own sexual attractiveness was reduced after exposure to Instagram content within the slim-athletic ideal in form of “fitspiration” pictures. Furthermore, the appreciation of one’s own body is considered a predictor of perceived own sexual attractiveness (Amos and Mccabe, 2015). Thus, it seems plausible that, in addition to social comparison with a similar group in terms of characteristics such as age and gender, comparative processes in relation to perceived sexual attractiveness, as well as the own assessment of one’s own sexual attractiveness can also play a role in one’s own body dissatisfaction.

There is meta-analytical evidence for the negative effects of exposure to objectifying media content in the context of hegemonic beauty ideals on aspects of body image such as body dissatisfaction in women (Grabe et al., 2008) and in men (Barlett et al., 2008), with mass media such as TV or magazines being addressed in these studies. In recent years, social media, especially image-based networks such as Instagram, have taken on an increasingly relevant role in the everyday lives of young people. For users under the age of 30, Instagram is the social medium with the highest daily usage rate in Germany (Beisch and Koch, 2021). This changes the way young people in particular are exposed to hegemonic beauty ideals in terms of quantity: people no longer need to purchase magazines or watch TV shows at certain times of the day to experience media exposure, but have continuous access to the installed app on their mobile phone, which is an algorithm-based endless source of visual content. Differences can also be seen in the quality of media content: in contrast to mass media, not only celebrities can be seen on Instagram, but to a large extent peers and influencers, who are often perceived as close and authentic (Tiggemann and Zaccardo, 2016; Lou and Yuan, 2019), which facilitates social comparisons. Furthermore, Instagram users are consumers at the same time and can share their own images and receive feedback in terms of interactions such as likes or comments. In terms of Bandura (1971) social cognitive learning theory, repeated exposure to images in the context of beauty ideals can lead to their imitation (Baumgartner-Hirscher and Zumbach, 2019), which is facilitated by a modified self-representation using the filters and image editing options freely available *via* Instagram, and lead to an easier consolidation of the ideal. The massive prevalence of the representation of hegemonic beauty ideals on Instagram, the slim-athletic ideal for women (Boepple and Thompson, 2016; Ahrens et al., 2022) and the muscularity ideal for men (Fatt et al., 2019), illustrates the number of posts shared (time July 2022) on hashtags categories such as #beauty (508 million), #model (348 million), #fitness (503 million), #fitspo (74.9 million), #muscle (68.4 million) or #thin, which has 2.8 million shared posts despite Instagram warning that it is a sensitive topic. By comparison, the most popular hashtag categories that advocate positive body image or body diversity have a much lower number of images, such as #bopo (1.3 million), #body positivity (10.5 million) or #normalize normal bodies (325,000).

Previous research on the effects of social media content on body dissatisfaction has placed a strong focus on the female gender, finding negative effects of exposure to content within the thin (slim) and fitspiration (lean-athletic) ideals on body dissatisfaction in women (Robinson et al., 2017; Cohen et al., 2019); while for men, shirtless images within the muscularity ideal had a particularly negative effect on body satisfaction (Tiggemann and Anderberg, 2020). Preliminary meta-analytic evidence speaks of a small negative effect of exposure to body-centered content within the beauty ideals *via* social media on body satisfaction (Saiphoo and Vahedi, 2019). Cohen et al. (2019) similarly found that exposure to Instagram content in the context of body positivity increased body satisfaction in a female sample. Body positivity Instagram content includes images of normal to overweight individuals, who advocate for a positive view of their own bodies (Cohen et al., 2021). Typically, Body Positivity images found on Instagram tend to show more diverse body images, mostly of women, with 94% of the content depicting normal to overweight bodies and is accompanied by quotes promoting body acceptance as well as the existence of beauty, regardless of physical dimensions (Cohen et al., 2021). Research has found positive effects of exposure to Body Positivity content on women (Cohen et al., 2019), nevertheless, the description of this content theme only represents a fraction of the Instagram consuming population, and the effect of the exposure to images cannot be separated from accompanying quotes. Because of that, in the present study a sample of visual stimuli in the framework of bodily diversity were presented focusing on a gender mixed image pool, whilst aiming variability of different aspects such as body shape and weight, the presence of body hair, acne, or physical disability. This diversity increased the possibility that study participants could identify similarities between their own appearance and that of the individuals pictured. Furthermore, references to an appraisal of beauty were avoided by foregoing the use of quotes. In a longitudinal study, Fioravanti et al. (2021a) found that daily exposure to body-positive images was associated with the highest positive trend in mood scores, whereas daily exposure to fitspiration imagery was associated with the highest negative trend in mood scores. The present study explores whether short term exposure to images in the context of hegemonic beauty ideals or in the context of body diversity can produce changes in the mood of the sample.

The aim of this contribution is to better understand the effects of hegemonic beauty vs. body diverse Instagram content in a ecologic valid setting, firstly by using an online survey that is easily accessible on mobile devices and thus avoiding laboratory specific interferences, and secondly through the gender-mixed visual stimuli. To best knowledge of the authors, no published experimental study in the subject area could be found, in which the participants in a gender heterogeneous sample were exposed to images of both their own and the opposite sex. The assumption that individuals active on Instagram only view photographs of their own sex, however, is questionable. Furthermore, the role of potential influential factors on body dissatisfaction is examined regarding potential moderation and mediation effects. Moreover, the current investigation aims to make a contribution to the concept of social media literacy in the sense of a critical engagement with beauty ideals portrayed in social media and the proposal of alternative content based on the stimuli from the control group, focused on depicting bodily diversity, without appraising a specific interaction with the own body, like it is intended in other content such as Body Positivity, and thus aiming a more neutral approach to body diversity.

The presented models, theories and considerations on the influence of different Instagram content on the body dissatisfaction of young women and men led to the establishment of the following research hypotheses:

Research question “Group differences”:

*H1: Compared to exposure to body diversity images, exposure to the hegemonic beauty ideal will have a negative effect on body satisfaction.*

*H2: Compared to exposure to body diversity images, exposure to the hegemonic ideal will have a negative effect on body satisfaction.*

Research question “Moderating and Mediating Constructs”:

*H3a: The tendency for upward social comparison with public self-attention moderates the effect of exposure to hegemonic beauty ideals vs. body diversity images on body dissatisfaction.*

*H3b: The internalization of the hegemonic beauty ideal moderates the effect of exposure to hegemonic beauty ideals vs. body diversity images on body dissatisfaction.*

*H3c: Comparative processes regarding sexual attractiveness mediate the influence of exposure to hegemonic beauty ideals vs. body diversity images on body dissatisfaction.*

*H3d: The assessment of one’s own sexual attractiveness mediates the influence of exposure to hegemonic beauty ideals vs. body diversity images on body dissatisfaction.*

In addition to the presented research hypotheses, further exploratory analyses were conducted. In evolutionary psychology, one’s assessment of the extent to which one is perceived as sexually attractive by one’s preferred sex is a predictor of the extent to which one is affected by information about one’s appearance derived from social comparisons (Bergstrom et al., 2004). Accordingly, the extent to which the assessment of one’s own sexual attractiveness in the sample studied could predicted the extent of comparison regarding sexual attractiveness and whether this tended to differ between groups was exploratively examined. Research on the influence of mass media on body dissatisfaction has identified associations between indicators of media exposure and negative effects on body image phenomena, for example, based on the number of hours of TV or magazine content consumed (Tiggemann, 2002). On this regard, exploratory analyses were conducted to determine whether the level of intensity with Instagram content as a predictor within multiple mediation models with parallel mediators predicted post-measurement body dissatisfaction in the sample studied.

## Methods and materials

### Sample

The study was designed as an online survey *via* the LimeSurvey platform, participation was possible *via* PC, tablet or mobile phone. The link to the survey was shared *via* the Stories feature of various Instagram profiles, which is how the majority of the sample was recruited. Further 22 participants were bachelor students of psychology from the University of Duisburg-Essen who were credited with subject hours. The remaining participants received no compensation for study participation. After entering the LimeSurvey link, the participants read a text which stated that the study was in the framework of a Master’s Thesis about photography in social media, they were informed about the duration of the survey and the contact data of the researcher. Furthermore, a data protection statement was presented. The participants were only able to click on the “next” button, and thus enter the study, after clicking on a box with the explicit consent form. All questions of the study were marked as compulsory questions, so that participation was only saved as successful if all questions were answered. An approximate study duration of 10 to 15 min was calculated. Based on a G*Power analysis (Faul et al., 2009), the sample should consist of at least 134 persons. The parameters defined in the analysis corresponded to a repeated-measures ANOVA for a within-between interaction, in which a power (1-*β*) of 0.95, an alpha error of 0.05, a small effect size *f* of 0.1579, a correlation between repeated measures of 0.5, and a correction for violation of the sphericity assumption of 1 were defined. The effect size selected here was guided by the meta-analysis of Saiphoo and Vahedi (2019), which found an average overall effect size *r* of 0.156 for a total of 63 studies, which converts to an effect size *f* of 0.1579 and represents a small, significant positive association between social media use and body image disorders.

The online study was started by 287 people, with a dropout rate of 61 people. The final sample thus consisted of 226 people, of which 186 were female (82.3%) and 40 male (17.7%). The allocation into experimental and control groups was done using a randomization function *via* LimeSurvey. The experimental group consisted of 121 persons (53.5% of the total sample) and the control group of 105 persons (46.5% of the total sample). The study participants were between 18 and 40 years old, the average age was *M* = 23.13 years (*SD* = 4.58), with 75% of the sample between 18 and 25 years old.

### Design

In order to test whether there are differences in body dissatisfaction after exposure to Instagram content with hegemonic beauty ideals compared to Instagram content with body diversity, it had to be determined whether the expression of body dissatisfaction changes after exposure to the different visual stimuli (experimental vs. control group). Subjects in the experimental group viewed 20 images of women and men within the exposure that were visually categorized as “hegemonic ideals of beauty.” In the control group, the exposure included 20 images of women and men within the category of “body diversity.” Repeated measures of body dissatisfaction were taken at two time points, once before exposure (pre-measurement) and once after (post-measurement). The Independent variables (IVs) were the measurement time points (two-tailed) and group membership (two-tailed). The dependent variable was the level of reported body dissatisfaction. It is therefore a two-factorial mixed design with repeated measures. Furthermore, the mood of the participants was surveyed before and after the exposure to examine potential effects of the exposure to the different image types on the state mood. In order to answer the question “moderating and mediating constructs,” the potential moderating effects of the constructs “internalization of beauty ideals” and “tendency to make upward social comparisons” in the relationship between the exposure to the hegemonic beauty ideal vs. body diversity images and body dissatisfaction was examined; whereas the constructs “Comparative process regarding sexual attractiveness “and “assessment of one’s own sexual attractiveness” were surveyed and their role as parallel mediators was examined within the framework of a multiple mediation model. In addition, for the purpose of explorative research, the individual intensity of engagement with Instagram content as well as the individual assessment of the relevance of Instagram use on psychological well-being were queried.

### Material

#### Visual stimuli

Two sets of stimulus materials were created to conduct the study. The image selection process consisted of two parts. First, the characteristics of the images for both groups were set for the control group, the image pool should consist of pictures showing women and men with diverse body shapes. In addition, diversity in terms of other physical features, such as skin blemishes, physical disability or presence of body hair was included in the control group’s set of images, as evidence suggests that beauty flaws affecting the skin can have a negative impact on body image (Koo and Yeung, 2002). The absence of body-centered citations was intended to achieve a body-neutral approach. For the experimental group, a set of visual stimuli was created consisting of photographs of women and men who embody the hegemonic gender-typical ideals of beauty. Photographs of the male gender presented men who had high muscle mass with low body fat, particularly in the upper body (Pope et al., 2001; Olivardia et al., 2004). Only images depicting shirtless men were used, as such images should have a particularly negative effect on men’s body satisfaction (Tiggemann and Anderberg, 2020). The visual stimuli consisting of images of the female gender depicted women who are considered slender within the ideal of beauty, albeit visibly athletic without being obviously muscular, who also look tall and young and have a full bust (Levine and Smolak, 2002; Watson et al., 2019). The images were selected using the hashtag search function on Instagram. Hashtags used in the image search of the experimental group included #fitspo, #fit, #model. For the control group, body diversity characteristics were gender matched, e.g., for the characteristic “physical disability” each a picture of a woman and a man with an amputated leg was shown, same applied for the characteristic “skin condition,” “high body mass,” and so on. Hashtags used in image searches for the control group included: #normalizenormalbodies, #bopo, #acne, #bodyhair, #bodydiversity. The respective owners of the photographs were asked for permission to use their photographs in the study *via* the direct message function of Instagram, and were informed about the nature of the study. From the initially selected pool of images, only those pictures whose owners agreed to were used as visual stimuli. In order to achieve the required number of stimuli, a part of the pictures was collected following the same characteristics-based approach from the image data-base “Pexels,” being license-free photographs.

The way in which the exposure was carried out was based on the study by Cohen et al. (2019). The subjects viewed 20 images, depending on whether they were assigned to the experimental or control group: 10 women’s photographs and 10 men’s photographs, either within the framework of hegemonic beauty ideals (athletic-slim for women and muscularity ideal for men) or within the framework of physical diversity (heterogeneity in terms of body shape, weight, skin, body hair and physical disability). Each image was presented for 10 s, resulting in approximately 3 min of exposure (200 s). The visual stimuli were presented in the typical Instagram frame, with the username, profile picture, number of likes and comments eliminated or made invisible. The visual stimuli were either taken from public Instagram profiles, after written permission from the persons depicted, or they were license-free photographs from the image database “Pexels,” which corresponded to the assumed visual characteristics of the respective group.

The photographs were always presented to the participants in the same order, so that in both groups a woman’s picture was presented alternately after a man’s picture. The order was not randomized so that at no point were two or more women’s or men’s pictures presented one after the other. We also refrained from showing any information about the pictures (number of likes or comments) in order to avoid possible anchor effects in the subjective interpretation of the pictures during the exposure: the interaction mechanisms on Instagram, such as *likes*, are considered a sign of popularity of the content or, in the case of a low number of likes, of unpopularity (Götz, 2019). The respective captions were not presented in order to control for possible distortions and to be able to attribute possible changes in the dependent variable (DV) to the isolated effect of the picture content. Care was taken not to use pictures of people who are well-known in Germany in order to avoid distorted attitudes of the test persons towards the pictures due to possible sympathies or antipathies towards the persons depicted. Instead, the images originating from Instagram were taken from public, non-internationally known profiles from the Spanish, Australian and American regions.

#### Measuring instruments

The demographic data collected were age and biological sex. We deliberately did not collect the Body Mass Index (BMI) of the study participants, although this is a frequently surveyed construct. However, BMI is a variable that can contribute to the continuation of stigmatization of different body sizes (Barron et al., 2021). Three visual analogue scales (VAS) with a range of 1 to 100 were used to assess the DV body dissatisfaction. Three visual analogue scales were used to assess body dissatisfaction. VAS are commonly used in body satisfaction research because they are time-efficient, recall of previous data is difficult, and the scales are sensitive to small changes in measurement repetitions (Fioravanti et al., 2021b). In addition, evidence exists for correlating of VAS on body satisfaction with more complex measures of body satisfaction (Heinberg and Thompson, 1995). Similar VAS items were used by Barron et al. (2021) and Slater et al. (2017) to assess state body satisfaction, showing adequate reliability and internal consistency. Three items were created with VAS to elicit the DV, with the phrases “I am dissatisfied with my overall appearance” (VAS “completely satisfied” to “completely dissatisfied”); “I would change aspects of my appearance” (VAS “change nothing at all” to “change a lot”); and “I am dissatisfied with my figure” (VAS “completely satisfied” to “completely dissatisfied”). To determine internal consistency in our sample, Cronbach’s alpha was calculated for both body dissatisfaction pre-measurement and body dissatisfaction post-measurement subscales. The internal consistency was high for the pre-measurement, with a Cronbach’s alpha =0.846 and excellent for the post measurement, with Cronbach’s alpha =0.918. The variable “state mood” was assessed before and after the exposure using a VAS with the value range 1 to 100, the wording “My current mood is…” and the poles “very bad” to “very good.”

The subscale of the “Sociocultural Attitudes Towards Appearance Questionnaire German Version” (SATAQ-G, Knauss et al., 2009) was used to record the construct “internalization of beauty ideals.” The SATAQ-G (Knauss et al., 2009) was validated on a German-speaking sample (*N* = 1,610, 49% female, aged 14–16 years). The internalization scale of the SATAQ-G consists of 6 items each for female subjects and 6 items each for male subjects (items 6, 7, 8, 9, 11, and 16 of the questionnaire). The items differ for the sexes only on the basis of the formulated beauty ideals (slimness ideal for women, muscularity ideal for men). The items are answered within the framework of a five-point Likert scale (“disagree” to “completely agree”), whereby high values are associated with a high internalization of the beauty ideal. Significant correlations between the internalization subscale and body dissatisfaction are found for both women and men (Knauss et al., 2009), which speaks for the convergent validity of the subscale. The internal consistency of the internalization subscale of the SATAQ-G is Cronbach’s alpha =0.88 for women and = 0.84 for men and can thus be classified as high (Knauss et al., 2009). For a better fit of the items to the topic under investigation, some formulations were adapted. For example, in the wording of item 6 of the SATAQ-G, “in magazines and on TV” was changed to “on Instagram,” in item 7, “on TV or in films” was changed to “in social media” and in item 9, “in music videos” was changed to “in social media.” In the sample studied, the gender-specific versions of the internalization scale of the SATAQ-G had high internal consistencies, with a Cronbach’s alpha =0.872 for the women’s version and = 0.870 for the men’s version.

To capture the construct “tendency to make upward social comparisons,” a translation of the Upward Physical Appearance Comparison Scale (O’Brien et al., 2009) was used. The UPACS measures the individual tendency to make upward comparisons regarding appearance in different contexts (O’Brien et al., 2009). Only the English-language version of the UPACS can be found, but this was validated on a sample (*N* = 224, 60% women) from an Australian university, so that the sample is similar to the present study’s sample in terms of age (*M* = 19.97, *SD* = 3.87, range 18–49 years) and cultural background with regard to media accessibility and hegemonic beauty ideals (89.9% of the validation sample Caucasian/White, Australian students, Western beauty ideals). The UPACS consists of 10 items that are answered within the framework of a five-point Likert scale (“strongly disagree” to “strongly agree”) and are formulated in a gender-neutral way. The UPACS has very good internal consistency (Cronbach’s alpha = 0.92) and good two-week retest reliability (*r* = 0.79), as well as good construct validity. In addition, factors such as age and BMI are not moderators (O’Brien et al., 2009). People who score low on UPACS should show less negative affect following exposure to idealized media content (O’Brien et al., 2009). To better fit the items to the topic under study, the original wording was slightly modified for two items. For example, in item 2 the word “magazine models” was changed to “Instagram models,” and in item 3 the words “models and film stars” were changed to “models or influencers.” In the sample studied, the UPACS scale version had excellent internal consistency, with a Cronbach’s alpha =0.914.

In order to capture the construct “Comparison processes regarding one’s own sexual attractiveness” with the categories “Comparative process regarding sexual attractiveness “and “Assessment of one’s own sexual attractiveness,” two items with visual analogue scales with the value range 1 to 100 were used. This type of construct measurement was preferred due to the advantages of VAS mentioned above and the lack of validated questionnaires that capture the constructs of interest. Although these are questions that may be shameful or reluctant to answer, it was decided not to add a “do not specify” answer alternative and instead to emphasize in the question wording that the answer should be spontaneous and that there are no right or wrong answers. Two items were created with the following formulations: “Some of the people depicted in the photographs might be sexually attracted to me” and “I appear sexually attractive to other people” (VAS “strongly disagree” to “strongly agree”). The internal consistency of the scale was high, Cronbach’s alpha =0.802.

Finally, the personal relationship to Instagram as a social medium was recorded. This was done by asking whether the participants thought that Instagram had an impact on their psychological well-being, with the answer options “Yes, mainly positive impact,” “Yes, mainly negative impact” or “No.” In addition, the intensity of personal engagement with Instagram content was recorded using a VAS with a value range of 1 to 100, with the wording “I engage with Instagram content…” and the anchors “hardly at all” to “very intensively.”

#### Procedure of the study

As part of the online recruitment process, prospective students could access the survey directly by clicking on the link and participate at any time during the 4 weeks of the recruitment process. They were informed that this was a 15-min online study in which 18 to 40 year-olds could participate anonymously from their mobile phone or PC, and that this was a study on the topic of images in social media. After reading the privacy statement and confirming their consent, they entered the study. Randomization into experimental or control groups was done through a randomization function that was invisible to the participants, and at no point did the participants know that the study followed an experimental design or that the participants were assigned to one of two groups. First, age and biological sex were collected, followed by mood and DV body dissatisfaction. Subsequently, the subjects filled out the UPACS and SATAQ questionnaires. This was followed by a three-minute exposure to the visual stimuli. Immediately afterwards, exposure-related comparison and competition processes regarding one’s own sexual attractiveness were assessed using two VAS, and the current mood and DV body dissatisfaction (post measurement) were queried again. Lastly, the personal reference to Instagram was surveyed. Finally, the participants were informed that the study had ended and were given contact details for the study management. The different sections of the study described above (privacy statement, demographics, pre-measure of mood, pre-measure of body dissatisfaction, SATAQ, UPACS, exposition, exposure-related comparison processes regarding one’s own sexual attractiveness, post-measure of mood, post-measure of body satisfaction, and personal engagement with Instagram) appeared only after the participants had completed the previous section, they were not able to scroll back to previous sections, and all the items were marked as mandatory questions.

## Results

### Demographic data and preliminary analysis

The average mood of the participants before exposure was *M* = 66.37, *SD* = 19.90, with higher values being associated with a better mood. In terms of individual intensity of engagement with Instagram content, this averaged *M* = 51.29, *SD* = 25.65 (scale 1 to 100), higher values represent higher intensity). The majority of the sample (*n* = 208; 92% of the total sample) indicated that Instagram use had a predominantly negative impact on psychological wellbeing, 12 individuals (5.3%) indicated that Instagram use had a predominantly positive impact on psychological wellbeing and 6 individuals (2.7%) indicated that Instagram use had no impact on psychological wellbeing.

Simple analyses of variance were performed to test for possible systematic differences between the groups. No significant differences were found between groups on age, *F*(1, 224) = 0.864, *p* = 0.354; gender, *F*(1, 224) = 0.041, *p* = 0.839; pre-measure of mood, *F*(1,224) = 1.425, *p* = 0.234; or intensity of engagement with Instagram content, *F*(1,224) = 1.338, *p* = 0.249. Furthermore, one way ANOVAS were performed to test for possible systematic differences between the groups on the UPACS and SATAQ-G scores, as well as on the assessment of one’s own sexual attractiveness. Homogeneity of variances was asserted using Levene’s Test which showed that equal variances could be assumed for the UPACS score; *p* = 0.363, the SATAQ-G score, *p* = 0.830 and the assessment of one’s own sexual attractiveness, *p* = 0.296. No significant differences emerged between the groups on the UPACS score, *F*(1, 224) = 2.78, *p* = 0.097; the SATAQ-G score, *F*(1, 224) = 0.363, *p* = 0.548; or the assessment of one’s own sexual attractiveness, *F*(1,224) = 0.417, *p* = 0.519. The groups did not differ significantly from each other on the pre-measure of body dissatisfaction, *F*(1,224) = 1.601, *p* = 0.207. Given the lack of systematic differences on demographic variables, the measured constructs or the pre-measure of DV body dissatisfaction between the groups, it can be assumed that the groups were comparable.

### Question “group differences”

#### Effect of image type on body dissatisfaction

To answer the question “group differences,” a mixed ANOVA with repeated measures was conducted. The between-subjects factor was group membership (experimental vs. control group), the within-subjects factor was time (pre- vs. post-measurement).

Mixed ANOVA revealed a statistically significant interaction between the measurement time points and the study groups, *F*(1, 224) = 30.202, *p* < 0.001, partial *η^2^* = 0.119. No simple main effects of the between-subjects factor were found. When tested for simple main effects of the within-subject factor, there was a statistically significant effect of time on body dissatisfaction in the experimental group, *F*(1, 120) = 9.275, *p* = 0.003, partial *η^2^* = 0.072. In the control group, there was a statistically significant effect of time on body dissatisfaction, *F*(1, 104) = 25.961, *p* = < 0.001, partial *η*^2^ = 0.200. There was no significant main effect of the variable time, *F*(1, 224) = 0.790, *p* = 0.375, partial *η*^2^ = 0.004. There was no significant main effect of group, *F*(1, 224) = 0.044, *p* = 0.835, partial *η^2^* =. 001. Furthermore, when looking at the descriptive statistics of the Mixed ANOVA, it can be seen that the significant results found run in a hypothesis-compliant direction: in the experimental group, body dissatisfaction increased in the pre-post comparison (*M*(Pre) = 43. 34; *SD* = 20.71, *M*(Post) = 46.97; *SD* = 25.27), while body dissatisfaction decreased in the pre-post comparison for the control group (*M*(Pre) = 47.04; *SD* = 23.28, *M*(Post) = 42.01; *SD* = 23.89; Table 1). Furthermore, in the descriptive statistics, there were no different tendencies depending on gender in either the experimental or the control group.

**Table 1:** Descriptive statistics of the mixed ANOVA.

Measurement time	Group	Sex	*M*	*SD*	*N*
Pre	EG	f	45,06	20,51	99
		m	35,59	20,28	22
		Total	43,34	20,71	121
	CG	f	47,67	23,71	87
		m	43,96	21,43	18
		Total	47,04	23,28	105
	Total		45,06	21,97	226
Post	EG	f	47,40	25,09	99
		m	45,02	26,58	22
		Total	46,97	25,27	121
	CG	f	43,46	23,81	87
		m	35,00	23,66	18
		Total	42,01	23,89	105
	Total		44,67	24,71	226

Value range 1 to 100, higher mean values are associated with higher body dissatisfaction (f, female; m, male).

Thus, H1 can be confirmed. Furthermore, a non-hypothesized effect can be reported, since the exposure of the control group to visual stimuli not only had no negative effect on body satisfaction, but also reduced body dissatisfaction in the pre-post comparison.

#### Effect of image type on mood

To assess pre-post differences in the state mood measure, a mixed ANOVA was conducted. Levene’s test showed that the variances for the post measurement of state mood were not equal between the groups, *F*(1, 224) = 7.725, *p* = 0.006. Therefore, the results of the conducted mixed ANOVA cannot be interpreted. Further mixed ANOVAS were conducted for each sex separately. For men, Levene’s test again showed that the variances for the post measurement of state mood were not equal between the groups, *F*(1, 38) = 6.761, *p* = 0.013, therefore the mixed ANOVA could not be interpreted.

For women, there was homogeneity of the error variances in the pre and post measurement of state mood, as assessed by Levene’s test (*p* > 0.05). For women, there was a statistically significant interaction between the measurement time points and the study groups, *F*(1, 184) = 15.047, *p* = < 0.001, partial *η^2^* = 0.076. There was a significant main effect for time, *F*(1, 184) = 18.527, *p* = < 0.001, partial *η^2^* = 0.091. There was no significant main effect for group, meaning that the study group did not differ significantly, *F*(1, 184) = 0.223, *p* = 0.683. When tested for simple main effects of the within-subject factor, there was a statistically significant effect of time on state mood in the experimental group, *F*(1, 98) = 28.286, *p* =. <0.001, partial *η^2^* = 0.224. In the control group, there was no statistically significant effect of time on state mood, *F*(1, 86) = 0.122, *p* = 728, partial *η*^2^ = 0.001. The descriptive statistics show that the significant results found for women in the experimental group run in a hypothesis-compliant direction.

Given that the mixed ANOVA could not be interpreted for the complete sample, but just for the female sample, the descriptive statistics on state mood are examined. Looking at the descriptive statistics, it can be seen that the mood of both sexes in the experimental group tended to worsen after the exposure. Descriptively, there were no gender differences; the pre-post difference was approx. 9 units for both genders. Descriptively, gender differences can be observed in the control group. On average, there were hardly any changes in the mood of the female subjects (*M* (pre) = 65.61; *SD* = 19.07, *M* (post) = 65.15; *SD* = 21.90), while the male subjects showed a slight increase in mood of about four units on average after exposure to the images of the category of physical diversity. Table 2 gives an overview of the parameters for mood pre- and post-measurement, divided by group and gender.

**Table 2:** Mood pre- and post-measurement, divided by group and gender.

Group	Sex	Mood	*M*	*SD*	*N*
EG	f	Pre	68,36	20,83	99
		Post	59,52	25,47	99
	m	Pre	65,50	21,48	22
		Post	56,55	27,25	22
CG	f	Pre	65,61	19,07	87
		Post	65,15	21,90	87
	m	Pre	60,17	16,34	18
		Post	64,94	18,42	18

Higher values are associated with a better mood. Value range 1 to 100, with the poles “bad” to “good.”

Thus, H2 can be confirmed for the female participants of the study. For male participants, the descriptive statistics run in a hypothesis-compliant direction, nevertheless, since the results of the mixed ANOVA could not be interpreted due to the significant Levene’s Test, no statements regarding the statistical significance of these results can be done.

### Question “influencing constructs”

#### Moderation analysis

A moderation analysis was conducted using the PROCESS macro by Hayes (2018) for SPSS, which uses ordinary least squares regression, yielding unstandardized coefficients for all effects. Bootstrapping with 5,000 samples together with heteroscedasticity consistent standard errors (HC3) were used to compute the confidence intervals. The moderation effects of the tendency to make upward social comparisons (UPACS score) and of the individual internalization of the gender-typical beauty ideal (SATAQ score) on the relationship between the exposure to images of the image types and body dissatisfaction were examined.

Regarding the UPACS score, a moderation analysis was conducted to determine whether the interaction between exposure to hegemonic beauty ideals vs. body diversity and the tendency to make upward social comparisons significantly predicted the change scores from pre- to post-measurement of body dissatisfaction. The overall model was significant, *F*(3, 222) = 11.427, *p* = <0.001, predicting 15.53% of the variance. The results of the moderation analysis show that the tendency to make upward social comparisons moderated the effect between the exposure content and the change scores in body dissatisfaction from pre- to post- exposure significantly, *ΔR^2^* = 1.97%, *F*(1,222) = 4.344, *p* = 0.038, 95% *CI* [0.443, 8.006].

Regarding the SATAQ-G score, a moderation analysis was conducted to determine whether the interaction between exposure to the hegemonic beauty ideals vs. body diversity and the internalization of the beauty ideals significantly predicts the change scores from pre- to post-measurement of body dissatisfaction. The overall model was significant, *F*(3, 222) = 12.826, *p* = <0.001, predicting 17.92% of the variance. The results of the moderation analysis show that the tendency to make upward social comparisons moderated the effect between the exposure content and the change scores in body dissatisfaction from pre- to post- exposure significantly, *ΔR^2^* = 4.73%, *F*(1,222) = 12.544, *p* = 0.0005, 95% *CI* [2.468, 8.362].

In sum, the conducted moderation analyses showed that the tendency to make upward social comparisons and the internalization of the beauty ideals, respectively, moderated the effect between the exposure content and the change scores in body dissatisfaction. Thus, hypotheses H3a and H3b can be confirmed.

### Mediation analysis

A mediation analysis was conducted using PROCESS macro version 4.0, which requires checking several prerequisites (Hayes, 2018). In the PROCESS macro, Model 4 was specified for mediation with multiple parallel mediation pathways, with group membership as the X variable, comparative process regarding sexual attractiveness and assessment of own sexual attractiveness as mediators (M1, M2), and post-measurement of body dissatisfaction as the Y variable. Furthermore, the number of bootstrap samples was set to 10,000 (Baltes-Götz, 2020). The interpretation of the mediation analysis was done in four steps, based on Hayes (2018). First, we checked whether there was a total effect in the model without the influence of the mediators. In a further step, the individual paths of the model were tested for significance to determine whether the IV predicted the individual mediators and whether the individual mediators predicted the DV. By taking the product of the paths, the indirect effects (the effects of the mediators) were calculated. This is the essential step to be able to prove mediation relations in the model. The last step was to check whether the effect of IV on DV was reduced from the model without mediators to the model with mediators, in order to determine whether mediation was complete (if the direct effect was not significant) or partial (if the direct effect was significant).

When looking at the total effect in the model without the influence of mediators, no statistically significant effect of group membership on the post-measurement of body dissatisfaction was found, *B* = − 4,961, *p* = 0.133. The negative coefficient means hereby that in the calculated mediation model, exposure to images in the control group compared to the reference group (the experimental group, dummy-coded with the number 1) reduced body dissatisfaction in the post-measurement by approximately 4.961 units, although the total effect was not significant.

After including mediators M1 and M2 in the model, the following effects were found: group membership significantly predicted interpersonal competition for sexual attractiveness, *B* = 18.871, *p* < 0.001. Assessment of one’s sexual attractiveness significantly predicted body dissatisfaction, *B* = −0.336, *p* = < 0.001. The direct effect (c’) in the model, was not statistically significant, *B* = −2.487, *p* = 0.449.

The indirect effects of the model represent the products of the individual paths of the mediators (path a x path b of the respective mediator). Thus, for M1 an effect of ab = −1.717 can be determined and for M2 ab = −0.753. The significance of the indirect effects can be determined using the bootstrap confidence intervals: if the lower and upper confidence intervals (BootLLCI and BootULCI) do not include 0, a significant indirect effect can be assumed. Since the bootstrap confidence intervals included 0 for both mediators, it can be assumed that the indirect effects in the model are not significant, even if individual indirect paths in the model show significant effects.

**Figure 1:**
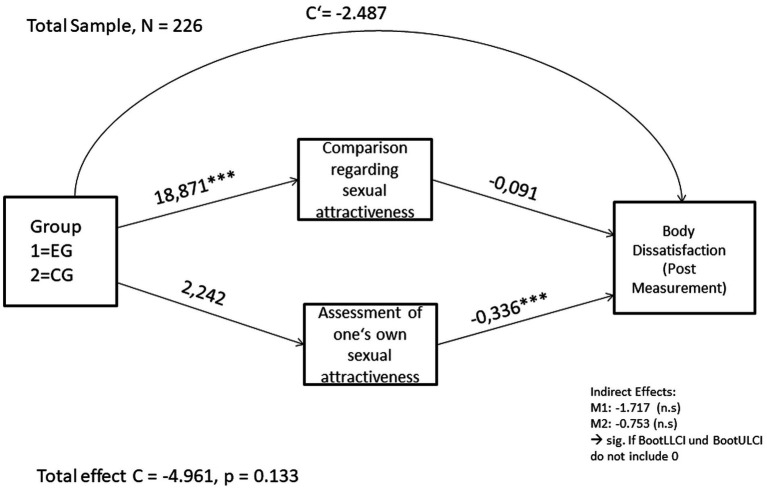
Calculated mediation model with direct effect and path effects. The asterisks behind the coefficients indicate the significance level: ***p < 0.001.

Thus, it can be concluded that the effect between exposure to images of hegemonic beauty ideals or images of diverse bodies on body dissatisfaction is only partially mediated by comparative process regarding sexual attractiveness and the assessment of one’s own sexual attractiveness. Thus, the hypotheses H3c and H3d could not be confirmed. Figure 1 gives an overview of the model.

### Explorative analysis 1

In addition to the hypotheses formulated *a priori*, two explorative analyses were conducted on the basis of the data collected. The first explorative analysis examined the extent to which the assessment of one’s own sexual attractiveness in the sample studied (M4 in the mediation model calculated *a priori*) could predict the extent of comparative processes with regard to sexual attractiveness (M3 in the mediation model calculated *a priori*) and whether these processes tended to differ between the groups. For this purpose, a linear regression was carried out for both the experimental and the control group, with M3 as DV and M4 as IV.

In the experimental group, the assessment of one’s own attractiveness explained 62.8% of the variance in response behavior. This proportion is significant, *F*(1, 119) = 77.53, *p* = < 0.001. For the experimental group, the assessment of one’s own sexual attractiveness significantly predicted how strongly participants indicated that the people depicted in the pictures could potentially be sexually attracted to them (*b* = 0.653, *β* = 0.628, *t* (119) = 8.805, *p* < 0.001).

In the control group, the assessment of one’s own attractiveness explained 76.3% of the variance in the criterion. This proportion is significant, *F*(1, 103) = 143.43, *p* < 0.001. For the control group, the assessment of one’s own sexual attractiveness significantly predicted how strongly participants indicated that the people depicted in the pictures could potentially be sexually attracted to them (*b* = 0.839, *β* = 0.763, *t*(103) = 11.976, *p* < 0.001).

These results suggest that in the sample studied, a higher assessment of one’s own sexual attractiveness held as a protective factor against comparative processes regarding sexual attractiveness. This effect was slightly higher in the control group: the one unit increase in the estimation of one’s own sexual attractiveness increased by 0.839 units the positive estimation of how the participants said the people depicted in the photos would perceive them. This protective effect was also observed in the experimental group, albeit slightly lower (*b* = 0.653).

### Explorative analysis 2

We explored whether the level of individual engagement with Instagram content as a predictor in multiple mediation models with parallel mediators predicted the post-measurement of body dissatisfaction in the sample studied. For this, two mediation models were computed using Hayes (2018) PROCESS macro *via* SPSS. The mediation models were calculated analogously to the mediation model set up in the context of the second research question, with the following differences: the intensity of engagement with Instagram content was specified as a predictor (X), the tendency to make upward social comparisons (UPACS score), the individual internalization of the gender-typical beauty ideal (SATAQ score), comparative process regarding sexual attractiveness and assessment of own sexual attractiveness were assessed as mediators (in the following analysis these variables are shortened as M1, M2, M3, and M4), and post-measurement of body dissatisfaction as the Y variable. Furthermore, separate mediation models were calculated for the experimental group and the control group, respectively.

First, the mediation model was calculated for the experimental group. A statistically significant effect of the intensity of engagement with Instagram content (abbreviated as “intensity” in the following) in the experimental group on the post-measurement of body dissatisfaction was found, *B* = 0.2553, *p* = 0.009.

After including mediators M1 to M4 in the model, the following effects were found in the paths: intensity significantly predicted the tendency to upward social comparison, *B* = 0.009, *p* = 0.0063, further it significantly predicted the internalization of the beauty ideal, *B* = 0.0125, *p* = 0.0002. The tendency to upward social comparison significantly predicted body dissatisfaction, *B* = 10. 084, *p* < 0.001. Internalization of the beauty ideal significantly predicted body dissatisfaction, *B* = 5.8924, *p* = 0.0002. Comparative process regarding sexual attractiveness significantly predicted body dissatisfaction, *B* = − 0.1403, *p* = 0.0494. Assessment of one’s own sexual attractiveness significantly predicted body dissatisfaction, *B* = −0.2088, *p* = 0.01.

When considering the direct effect (c’) in the model, no statistically significant effect of intensity of engagement with Instagram content on the post-measurement of body dissatisfaction was found, *B* = 0.0886, *p* = 0.1963.

**Figure 2:**
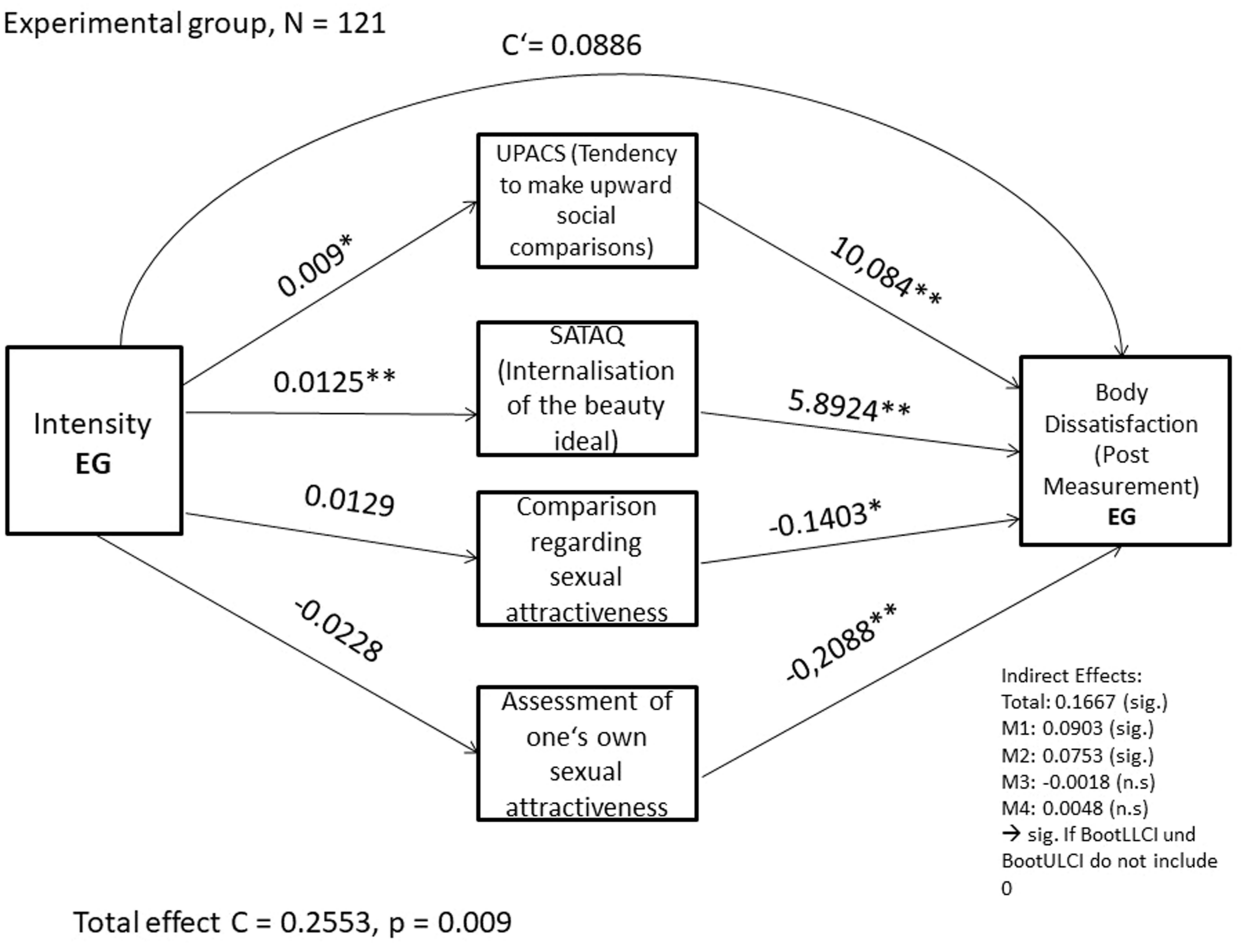
Calculated mediation model for the experimental group. The asterisks behind the coefficients indicate the significance level: *p < 0.05, **p < 0.01.

Looking at the indirect effects in the model, we found an effect of ab = 0.0903 for M1, ab = 0.0753 for M2, ab = −0.0018 for M3 and ab = 0.0048 for M4. Since for M1 and M2 the bootstrap confidence intervals did not include 0, it can be assumed that the indirect effects of the tendency to make upward social comparisons and the internalization of the beauty ideal significantly mediated the effect of the intensity of engagement with Instagram content on the post-measurement of body dissatisfaction in the experimental group. Figure 2 provides an overview of the mediation model for the experimental.

Furthermore, the mediation model was calculated for the control group. The total effect in the model was statistically significant, *B* = 0.3245, *p* = 0.002.

After including mediators M1 to M4 in the model, the following effects were found in the paths: intensity significantly predicted the tendency to make upward social comparisons, *B* = 0.0106, *p* = 0.0023, which in turn significantly predicted body dissatisfaction, *B* = 8. 1712. Furthermore, intensity significantly predicted the internalization of the beauty ideal, *B* = 0.0121, *p* = 0.0009. Lastly, the assessment of one’s own sexual attractiveness significantly predicted body dissatisfaction, *B* = −0.3106, *p* = 0.0139.

When looking at the direct effect (c’) in the model, a statistically significant effect of intensity of engagement with Instagram content on the post-measure of body dissatisfaction was found, *B* = 0.2132, *p* = 0.0085.

**Figure 3:**
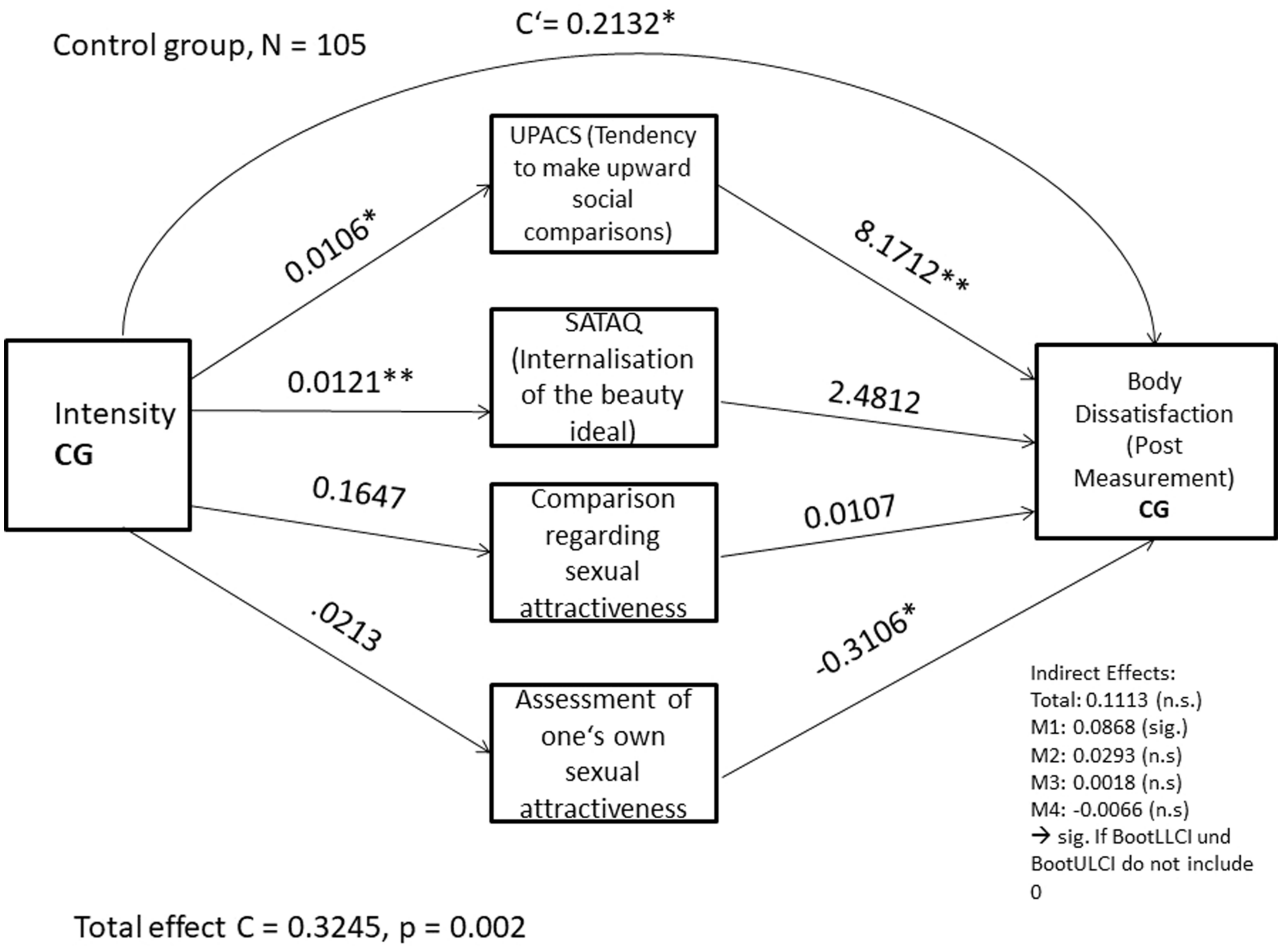
Calculated mediation model for the control group. The asterisks behind the coefficients indicate the significance level: *p < 0.05, **p < 0.01.

With regard to the indirect effects in the model, for M1 there is an effect of ab = 0.0868, for M2 ab = 0.0293, for M3 ab = 0.0018 and for M4 ab = −0.0066. Since only for M1 the bootstrap confidence intervals do not include 0, it can be assumed that the indirect effect of the tendency for upward social comparisons in the model significantly mediated the effect of the intensity of engagement with Instagram content on the post measurement of body dissatisfaction in the control group. Figure 3 provides an overview of the mediation model for the control group.

When looking at the two calculated mediation models, several interesting effects can be observed. Significant total effects of the model are found in both the experimental and control groups, suggesting that regardless of the content of the images viewed, the individual intensity of engagement with Instagram content has an effect on the post measurement of body dissatisfaction. In the experimental group, this effect disappeared after the mediators were included in the model (the direct effect was not significant), which speaks for full mediation. In particular, the indirect effects of M1 and M2 were significant in the experimental group, although the pathways of M3 and M4 significantly predicted body dissatisfaction. For the control group, the effect of X on Y did not disappear after inclusion of the mediators (the direct effect was still significant), and only the indirect effect of M1 was significant, although M2 was also significantly predicted by the intensity of engagement with Instagram content, and M4 significantly predicted body dissatisfaction. Thus, it can be assumed that in the control group, the effect of the intensity of engagement with Instagram content was only partially mediated by the tendency to make upward social comparisons. Based on these results, with regard to the sample studied, it can be concluded that the intensity of engagement with Instagram content had a small effect on body dissatisfaction independent of the images observed, and that this effect was fully mediated by multiple mediators in the experimental group. However, as this is an exploratory, post-hoc analysis, the reported results can only be considered hypothesis-generating, and thus are only meant to stimulate further research.

## Discussion

This study investigated the effects of Instagram content in the context of hegemonic beauty ideals in the experimental group and body diversity in the control group on body dissatisfaction in a sample of young women and men, examined the effect of exposure on the state mood of the participants as well as possible moderating and mediating constructs. In line with the hypothesis, a statistically significant interaction between the measurement points and the study groups was found within the framework of the research question on group differences regarding body dissatisfaction. Furthermore, in line with the hypothesis, there was a significant increase in body dissatisfaction after exposure in the experimental group and, extending the hypothesis, a reduction in body dissatisfaction after exposure in the control group. The hypothesis regarding the effect of exposure to the images in the experimental group on mood could be confirmed for women, with a deterioration of the state mood after exposure in the experimental group, whilst no change was observed in the control group; for men, the mixed ANOVA could not be interpreted, nevertheless the descriptive statistics on mood did run in a hypothesis-compliant direction. The two hypotheses regarding moderating effects of the tendency to make upward social comparisons as well as the internalization of the gender specific beauty ideals on the effect between image type exposure and body dissatisfaction could be confirmed. The hypotheses formulated for the presumed mediating constructs could not be confirmed within the framework of the study, as the model examined did not yield any significant mediation, although significant correlations between the model components were found: group membership predicted comparative processes regarding sexual attractiveness, whereas the assessment of the own sexual attractiveness predicted the post-exposure body dissatisfaction. Two exploratory analyses were conducted. The first showed that a higher assessment of one’s own sexual attractiveness had a protective effect on comparative processes in this regard in both groups, although this effect was somewhat higher in the control group. The second exploratory analysis revealed that the intensity of engagement with Instagram content had a small effect on body dissatisfaction in both groups, and that this effect was mediated by the mediators studied only in the experimental group.

The study conducted extends previous empirical findings regarding the effects of different Instagram content on body dissatisfaction in three ways in particular. The study design, which is based on the study by Cohen et al. (2019) with regard to the exposure duration of approx. Three minutes with 20 pictures per study group, can be considered ecologically valid, as participation in the study could be analogous to usual Instagram use, since the online conception made the survey possible *via* the preferred own device (mobile phone, tablet or PC), and laboratory or experimenter effects were thereby excluded. Previous studies examining similar research topics tend to follow correlational (e.g., Fatt et al., 2019) or laboratory experimental designs (e.g., Brown and Tiggemann, 2016; Robinson et al., 2017; Cohen et al., 2019). Furthermore, the sample consisted of both women and men, and all participants were exposed to the same photographs that also depicted women and men. The majority of publications on the effects of social media on body dissatisfaction examined female samples, although Instagram is an app that is used equally by women and men (Sumter et al., 2022). The studies published so far examine exposure to images of one’s own gender, both in female (e.g., Cohen et al., 2019; Munsch et al., 2021) and male samples (e.g., Tiggemann and Anderberg, 2020; Sumter et al., 2022). A design in which a person is exposed to images of their own gender is consistent with Festinger’s theory of social comparisons (1954), on the basis of which comparison is sought with people who share characteristics such as age or gender. On Instagram, exposure to images of both sexes is the norm, unlike previous mass media, such as magazines, which had to be purchased individually and were often strictly gender segregated. Based on the assumption that what the preferred gender perceives as sexually attractive also has an impact on body dissatisfaction (Bergstrom et al., 2004), and on the assumption of objectification theory (Fredrickson and Roberts, 1997) that people view themselves from an observer’s perspective, the chosen cross-gender visual stimuli allow for a wider range of possible social comparisons that also approximate the reality of Instagram content. A third way in which the study complements the research is in the visual stimuli chosen for the control group, which included images in the context of body diversity in terms of the variables of gender, body shape, weight, body hair, skin characteristics such as acne or vitiligo, and physical disability, without emphasizing an intentional salience of the desired effects of the images on body satisfaction through, for example, quotes, as is often the case with Body Positivity content (Cohen et al., 2021).

### Practical implications

The increase in body dissatisfaction observed in the experimental group after exposure to visual stimuli in the context of the representation of hegemonic beauty ideals *via* Instagram content supports the findings of previous, thematically related research and underlines the relevance of the influence of social media such as Instagram on young adults’ body dissatisfaction. Prevention measures are particularly relevant in a psychoeducational context. For this, the importance of social media literacy (SML for short) is emphasized. The concept of SML forms an extension of mass media-related media literacy (ML for short), refers to the ability to critically engage with and evaluate shared content in social media and is considered a protective factor against media influence on body image-related phenomena such as body dissatisfaction (Primack et al., 2009; Paxton et al., 2022). The influencing effects on body dissatisfaction by the tendency towards upward social comparisons and internalization of beauty ideals found is consistent with the mechanisms of action of ML in the prevention of body dissatisfaction: the presence of ML can resolve the pathway of media-generated pressure on the internalization of beauty ideals and the tendency towards appearance-related comparisons (McLean et al., 2016). Thus, a critical evaluation of ideals portrayed in the media and the knowledge that such content often does not correspond to reality, but is retouched, filtered or edited, can lead to the ideals being perceived as less plausible and desirable (McLean et al., 2016). The results of the current study regarding the effects of the different image contents on the state body dissatisfaction after a short exposure time can be used to promote the stated critical engagement specifically with Instagram content. Although empirical evidence for the protective role of SML on body dissatisfaction can already be found, the number of published studies is small. Previous SML research has focused particularly on prevention interventions among adolescents, where most effects can be found among young girls (Paxton et al., 2022). Given that the results found in the current study come from a gender heterogeneous sample of young adults, it seems reasonable to extend SML research to such target groups.

Another component of the study results that may be important for practical implications is the observed reduction in body dissatisfaction in the control group after exposure to content within the framework of bodily diversity. The reported reduction in body dissatisfaction after the three-minute exposure may reinforce the suggestion to consider photographs in the context of bodily diversity as another possibility of alternative Instagram content that can have a positive effect on aspects of psychological well-being. This can be actively sought by individual Instagram users and promoted in psychoeducational contexts, since as discussed in the introduction section, bodily diversity is not the kind of content mainly shown to the broad public in social media. Furthermore, these results underline the relevance of addressing bodily diversity in psychoeducational contexts and are in line with SML to promote a critical engagement with media portrayed and often distorted or retouched body representations.

### Limitations and recommendations for future studies

The study conducted is subject to several limitations. A cross-sectional design was used, with an exposure duration of approximately 3 min. Future studies should use longitudinal designs to examine long-term effects of exposure to different image content on body dissatisfaction. In the sample, 82% of the participants were female, while approximately 18% were male (40 out of a total of 226 subjects). Against this background, conclusions regarding gender comparisons are only possible to a limited extent, as the male sample in particular is not very representative. Furthermore, the variable gender was kept dichotomous and related to the biological sex, so that no information can be given on transsexual and intersexual or non-binary persons. The demographics assessed in this study included only the biological sex and age of the participants. Assessing further demographics such as educational level, sexuality, BMI or ethnicity will provide a greater predictive value to future studies, and constitutes a limitation to the current investigation. Future studies should include more representative samples in terms of sample size, gender distribution and assessed demographics to extend the findings. In order to economize the study in terms of time and content, an attempt was made to keep the constructs collected to a minimum. Constructs that could theoretically play a role in the topic under investigation, such as self-objectification, were not collected. The constructs “Comparative process regarding sexual attractiveness “and “assessment of one’s own sexual attractiveness” were each surveyed using a single item due to the lack of validated measurement instruments to examine the constructs of interest. For this reason, the results concerning these constructs should be interpreted with caution. The images used for the exposition were selected following the descriptive characteristics of the beauty ideals described in the literature as well as for different features of body diversity. Nonetheless, since an evaluation of the visual stimuli by independent judges did not take place and inter-rater reliability was not calculated, the possibility of bias can not be fully excluded. Furthermore, given that only 20 pictures per group were used for the exposition, the broad spectrum of bodily diversity could not be depicted.

Future studies should address desirably the limitations of the current study as stated above. Thus, a replication of the results with a larger sample, especially with a higher proportion of male subjects, a broader list of demographics as well as including validated measures of constructs regarding sexual attractiveness, would increase the quality of the study. Furthermore, future studies should try to assess which kind of social comparisons processes are more prevalent in a mixed sample when exposing to mixed stimuli. Additionally, and as stated before, the 20 pictures used to show body diversity cannot depict the broad spectrum of different bodies and their characteristics. Future studies should include a larger amount of stimuli in order to reach a broader range of body diversity. Moreover, to avoid bias, the image selection process should be enhanced by rating the stimuli by multiple independent judges.

### Conclusion

The presented study dealt with the social medium Instagram as a socio-cultural influencing factor on young adults’ body dissatisfaction. The effects of a three-minute exposure to image content within the framework of hegemonic beauty ideals and body diversity on body dissatisfaction were examined. Despite several limitations, the current study provides evidence on the detrimental effects of short term exposure to hegemonic beauty ideals on Instagram on body satisfaction and state mood, as well as on the positive effects of short term exposure to bodily diversity on body satisfaction. Furthermore, significant moderation effects were found. Moreover, despite the conducted mediation model not showing a significant mediation as predicted, the significant correlations between the explored model components, the reported moderating role of upward comparative tendencies and internalization of beauty ideals, as well as the associations found in the exploratory analyses suggest that the constructs observed can be influencing factors on body dissatisfaction. The current study can inspire future investigations on the role of the presented constructs and body diversity Instagram content in body dissatisfaction, whilst underlining the relevance of addressing body diversity and a critical engagement with portrayed beauty ideals in social media for the individual user and in psychoeducational contexts addressing young adults.

## Data availability statement

The raw data supporting the conclusions of this article will be made available by the authors, without undue reservation.

## Ethics statement

Ethical approval was not provided for this study on human participants because was not necessary according to the ethics committee. The patients/participants provided their written informed consent to participate in this study.

## Author contributions

RC planned and conducted the study. GS supervised the study and analyses and helped to set the manuscript. All authors contributed to the article and approved the submitted version.

## Conflict of interest

The authors declare that the research was conducted in the absence of any commercial or financial relationships that could be construed as a potential conflict of interest.

## Publisher’s note

All claims expressed in this article are solely those of the authors and do not necessarily represent those of their affiliated organizations, or those of the publisher, the editors and the reviewers. Any product that may be evaluated in this article, or claim that may be made by its manufacturer, is not guaranteed or endorsed by the publisher.

## References

[ref1] Ahrens J. Brennan F. Eaglesham S. Buelo A. Laird Y. Manner J.. (2022). A longitudinal and comparative content analysis of Instagram fitness posts. Int. J. Environ. Res. Public Health 19:6845. doi: 10.3390/ijerph19116845, PMID: 35682428 PMC9180174

[ref63] Amos N. McCabe M. P. (2015). Conceptualizing and measuring perceptions of sexual attractiveness: Are there differences across gender and sexual orientation? Personality and Individual Differences 76, 111–122. doi: 10.1016/j.paid.2014.11.057, PMID: 25778714

[ref2] Baltes-Götz B. (2020). Mediator- und Moderatoranalyse mit SPSS und PROCESS: Zentrum für Informations-, Medien- und Kommunikationstechnologie (ZIMK) an der Universität Trier (*mediator and moderator analysis with SPSS and PROCESS*). Trier: Centre for Information, Media and Communication Technology (ZIMK) at the University of Trier. Available online at: https://www.uni-trier.de/fileadmin/urt/doku/medmodreg/medmodreg.pdf

[ref3] Bandura A. (1971). Social learning theory. New York, NY: General Learning Press.

[ref4] Barlett C. P. Vowels C. L. Saucier D. A. (2008). Meta-analyses of the effects of media images on men’s body-image concerns. J. Soc. Clin. Psychol. 27, 279–310. doi: 10.1521/jscp.2008.27.3.279

[ref5] Barron A. M. Krumrei-Mancuso E. J. Harriger J. A. (2021). The effects of fitspiration and self-compassion Instagram posts on body image and self-compassion in men and women. Body Image 37, 14–27. doi: 10.1016/j.bodyim.2021.01.003, PMID: 33556914

[ref6] Baumgartner-Hirscher N. Zumbach J. (2019). Die Auswirkungen medialer Angebote auf das Körperbild von Jugendlichen: Eine experimentelle Studie mit impliziten und expliziten Methoden (the effects of media offerings on the body image of adolescents: an experimental study using implicit and explicit methods) Medien Pädagogik: Zeitschrift für Theorie und Praxis der Medienbildung, 37–60 doi: 10.21240/mpaed/00/2019.10.15.X.

[ref7] Beisch N. Koch W. (2021). 25 Jahre ARD/ZDF-Onlinestudie: Unterwegsnutzung steigt wieder und streaming/Mediatheken Sind weiterhin Treiber des medialen internets (25 years of the ARD/ZDF online study, mobile use on the rise again and streaming/media libraries continue to drive the media internet). Media Perspektiven 10, 486–503.

[ref8] Bergstrom R. L. Neighbors C. Lewis M. A. (2004). Do men find “bony” women attractive? Body Image 1, 183–191. doi: 10.1016/s1740-1445(03)00025-1, PMID: 18089150

[ref9] Blake C. (2015). “Definition des Begriffs Körperzufriedenheit” in *Wie mediale Körperdarstellungen die Körperzufriedenheit beeinflussen*. Definition of the term body satisfaction” in How media representations of the body influence body satisfaction (Wiesbaden: Springer VS). doi: 10.1007/978-3-658-07750-1_2

[ref10] Boepple L. Thompson J. K. (2016). A content analytic comparison of fitspiration and thinspiration websites. Int. J. Eat. Disord. 49, 98–101. doi: 10.1002/eat.22403, PMID: 25778714

[ref11] Brechan I. Kvalem I. L. (2015). Relationship between body dissatisfaction and disordered eating: mediating role of self-esteem and depression. Eat. Behav. 17, 49–58. doi: 10.1016/j.eatbeh.2014.12.008, PMID: 25574864

[ref12] Brennan M. A. Lalonde C. E. Bain J. L. (2010). Body image perceptions: do gender differences exist? Psi Chi. J. Psychol. Res. 15, 130–138. doi: 10.24839/1089-4136.jn15.3.130

[ref13] Brown Z. Tiggemann M. (2016). Attractive celebrity and peer images on Instagram: effect on women’s mood and body image. Body Image 19, 37–43. doi: 10.1016/j.bodyim.2016.08.007, PMID: 27598763

[ref14] Bucchianeri M. M. Arikian A. J. Hannan P. J. Eisenberg M. E. Neumark-Sztainer D. (2013). Body dissatisfaction from adolescence to young adulthood: findings from a 10-year longitudinal study. Body Image 10, 1–7. doi: 10.1016/j.bodyim.2012.09.001, PMID: 23084464 PMC3814026

[ref15] Cameron E. Ward P. Mandville-Anstey S. A. Coombs A. (2019). The female aging body: a systematic review of female perspectives on aging, health, and body image. J. Women Aging 31, 3–17. doi: 10.1080/08952841.2018.1449586, PMID: 29558298

[ref16] Cohen R. Fardouly J. Newton-John T. Slater A. (2019). #BoPo on Instagram: an experimental investigation of the effects of viewing body positive content on young women’s mood and body image. New Media Soc. 21, 1546–1564. doi: 10.1177%2F1461444819826530

[ref17] Cohen R. Newton-John T. Slater A. (2021). The case for body positivity on social media: perspectives on current advances and future directions. J. Health Psychol. 26, 2365–2373. doi: 10.1177%2F1359105320912450, PMID: 32191132 10.1177/1359105320912450

[ref18] Davidson T. E. McCabe M. P. (2005). Relationships between men’s and women’s body image and their psychological, social, and sexual functioning. Sex Roles 52, 463–475. doi: 10.1007/s11199-005-3712-z

[ref19] Esnaola I. Rodríguez A. Goñi A. (2010). Body dissatisfaction and perceived sociocultural pressures: gender and age differences. Salud Ment., 33, 21–29. Available online at: http://www.revistasaludmental.com/index.php/salud_mental/article/view/1333

[ref20] Fatt S. J. Fardouly J. Rapee R. M. (2019). # Malefitspo: links between viewing fitspiration posts, muscular-ideal internalization, appearance comparisons, body satisfaction, and exercise motivation in men. New Media Soc. 21, 1311–1325. doi: 10.1177%2F1461444818821064

[ref21] Faul F. Erdfelder E. Buchner A. Lang A. G. (2009). Statistical power analyses using G* power 3.1: tests for correlation and regression analyses. Behav. Res. Methods 41, 1149–1160. doi: 10.3758/BRM.41.4.1149, PMID: 19897823

[ref22] Festinger L. (1954). A theory of social comparison processes. Hum. Relat. 7, 117–140. doi: 10.1177/001872675400700202

[ref23] Fioravanti G. Svicher A. Ceragioli G. Bruni V. Casale S. (2021a). Examining the impact of daily exposure to body-positive and fitspiration Instagram content on young women’s mood and body image: an intensive longitudinal study. New Media Soc. doi: 10.1177%2F14614448211038904

[ref24] Fioravanti G. Tonioni C. Casale S. (2021b). # Fitspiration on Instagram: the effects of fitness-related images on women’s self-perceived sexual attractiveness. Scand. J. Psychol. 62, 746–751. doi: 10.1111/sjop.12752, PMID: 34170526 PMC8518738

[ref25] Fiske L. Fallon E. A. Blissmer B. Redding C. A. (2014). Prevalence of body dissatisfaction among United States adults: review and recommendations for future research. Eat. Behav. 15, 357–365. doi: 10.1016/j.eatbeh.2014.04.010, PMID: 25064281

[ref26] Fitzsimmons-Craft E. E. Bardone-Cone A. M. (2012). Examining prospective mediation models of body surveillance, trait anxiety, and body dissatisfaction in African American and Caucasian college women. Sex Roles 67, 187–200. doi: 10.1007/s11199-012-0151-5

[ref27] Fredrickson B. L. Roberts T. A. (1997). Objectification theory: toward understanding women’s lived experiences and mental health risks. Psychol. Women Q. 21, 173–206. doi: 10.1111/j.1471-6402.1997.tb00108.x

[ref28] Götz M. (2019). “Man braucht ein perfektes Bild” in Die Selbstinszenierung von Mädchen auf Instagram, “One needs a perfect picture” The self-presentation of girls on Instagram, Televizion Digital, vol. 1, 9–20.

[ref29] Grabe S. Ward L. M. Hyde J. S. (2008). The role of the media in body image concerns among women: a meta-analysis of experimental and correlational studies. Psychol. Bull. 134, 460–476. doi: 10.1037/0033-2909.134.3.460, PMID: 18444705

[ref30] Griffiths S. Hay P. Mitchison D. Mond J. M. McLean S. A. Rodgers B.. (2016). Sex differences in the relationships between body dissatisfaction, quality of life and psychological distress. Aust. N. Z. J. Public Health 40, 518–522. doi: 10.1111/1753-6405.12538, PMID: 27372301

[ref31] Grogan S. (1999). Body image: Understanding body dissatisfaction in men, women, and children. London; New York, NY: Routledge.

[ref32] Hayes A. F. (2018). The PROCESS macro for SPSS and SAS version 3.0 [computer software]. Available online at: https://afhayes.com.

[ref64] Heinberg L. J. Thompson J. K. (1995). Body image and televised images of thinness and attractiveness: A controlled laboratory investigation. J Soci. Clini. Psychol. 14, 325–338. doi: 10.1521/jscp.1995.14.4.325, PMID: 27598763

[ref33] Knauss C. Paxton S. J. Alsaker F. D. (2009). Validation of the German version of the sociocultural attitudes towards appearance questionnaire (SATAQ-G). Body Image 6, 113–120. doi: 10.1016/j.bodyim.2009.01.002, PMID: 19244000

[ref70] Koo J. Y. M. Yeung J. (2002). “Body image issues in dermatology” in Body image: A handbook of theory, research, and clinical practice: A handbook of science, practice, and prevention. eds. Cash T. F. Pruzinsky T. (Hrsg.) (Guilford Publications), S335–341. PMID:

[ref34] Lee-Won R. J. Joo Y. K. Baek Y. M. Hu D. Park S. G. (2020). Obsessed with retouching your selfies? Check your mindset!: female Instagram users with a fixed mindset are at greater risk of disordered eating. Personal. Individ. Differ. 167:110223. doi: 10.1016/j.paid.2020.110223

[ref200] Levine M. P. Smolak L. (2002). “Ecological and activism approaches to the prevention and change of negative body image” in Body image: A handbook of theory, research, and clinical practice: A handbook of science, practice, and prevention. eds. Cash T. F. Pruzinsky T. (Hrsg.) (Guilford Publications), S497–508., PMID:

[ref35] López-Guimera G. Levine M. P. Sánchez D. Fauquet J. (2010). Influence of mass media on body image and eating disordered attitudes and behaviors in females: a review of effects and processes. Media Psychol. 13, 387–416. doi: 10.1080/15213269.2010.525737

[ref36] Lou C. Yuan S. (2019). Influencer marketing: how message value and credibility affect consumer trust of branded content on social media. J. Interact. Advert. 19, 58–73. doi: 10.1080/15252019.2018.1533501

[ref37] Margraf J. Schneider S. (2018). “Lehrbuch der Verhaltenstherapie, Band 1: Grundlagen und Verfahren” in Textbook of behavior therapy, Fundamentals and Procedures, vol. 1 (Heidelberg: Springer)

[ref38] Martin A. Buhlmann U. (2020). Körperdysmorphe Störung und Körperunzufriedenheit (body dysmorphic disorder and body dissatisfaction). Psychotherapeut 65, 67–70. doi: 10.1007/s00278-020-00407-z

[ref39] McLean S. A. Paxton S. J. (2019). Body image in the context of eating disorders. Psychiatr. Clin. N. Am. 42, 145–156. doi: 10.1016/j.psc.2018.10.006 30704635

[ref40] McLean S. A. Paxton S. J. Wertheim E. H. (2016). The role of media literacy in body dissatisfaction and disordered eating: a systematic review. Body Image 19, 9–23. doi: 10.1016/j.bodyim.2016.08.002, PMID: 27572000

[ref41] Munsch S. Messerli-Bürgy N. Meyer A. H. Humbel N. Schopf K. Wyssen A.. (2021). Consequences of exposure to the thin ideal in mass media depend on moderators in young women: an experimental study. J. Abnorm. Psychol. 130, 498–511. doi: 10.1037/abn0000676, PMID: 34472886

[ref42] Noles S. W. Cash T. F. Winstead B. A. (1985). Body image, physical attractiveness, and depression. J. Consult. Clin. Psychol. 53, 88–94. doi: 10.1037/0022-006X.53.1.88 3980834

[ref43] O’Brien K. S. Caputi P. Minto R. Peoples G. Hooper C. Kell S.. (2009). Upward and downward physical appearance comparisons: development of scales and examination of predictive qualities. Body Image 6, 201–206. doi: 10.1016/j.bodyim.2009.03.003, PMID: 19447692

[ref01] Olivardia R. Pope H. G. Jr. Borowiecki J. J. III. Cohane G. H. (2004). Biceps and body image: The relationship between muscularity and self-esteem, depression, and eating disorder symptoms. Psychol. Men Mascul. 5, 112–120. doi: 10.1037/1524-9220.5.2.112, PMID: 27352102

[ref44] Paxton S. J. McLean S. A. Rodgers R. F. (2022). “My critical filter buffers your app filter”: social media literacy as a protective factor for body image. Body Image 40, 158–164. doi: 10.1016/j.bodyim.2021.12.009, PMID: 34968853

[ref02] Pope H. G. Jr. Olivardia R. Borowiecki J. Cohane G. (2001). The growing commercial value of the male body: A longitudinal survey of advertising in women’s magazines. Psychotherapy and Psychosomatics 70, 189–192. doi: 10.1159/000056252, PMID: 11408837

[ref45] Potter B. Pederson L. Chan S. Aubut J. A. Koval J. (2004). Does a relationship exist between body weight, concerns about weight, and smoking among adolescents? An integration of the literature with an emphasis on gender. Nicotine Tob. Res. 6, 397–425. doi: 10.1080/14622200410001696529, PMID: 15203775

[ref46] Primack B. A. Swanier B. Georgiopoulos A. M. Land S. R. Fine M. J. (2009). Association between media use in adolescence and depression in young adulthood. Arch. Gen. Psychiatry 66, 181–188. doi: 10.1001/archgenpsychiatry.2008.532, PMID: 19188540 PMC3004674

[ref47] Ridolfi D. R. Crowther J. H. (2013). The link between women’s body image disturbances and body-focused cancer screening behaviors: a critical review of the literature and a new integrated model for women. Body Image 10, 149–162. doi: 10.1016/j.bodyim.2012.11.003, PMID: 23265838

[ref48] Robinson L. Prichard I. Nikolaidis A. Drummond C. Drummond M. Tiggemann M. (2017). Idealized media images: the effect of fitspiration imagery on body satisfaction and exercise behavior. Body Image 22, 65–71. doi: 10.1016/j.bodyim.2017.06.001, PMID: 28654826

[ref49] Saiphoo A. N. Vahedi Z. (2019). A meta-analytic review of the relationship between social media use and body image disturbance. Comput. Hum. Behav. 101, 259–275. doi: 10.1016/j.chb.2019.07.028

[ref010] Slater A. Varsani N. Diedrichs P. C. (2017). #fitspo or #loveyourself? The impact of fitspiration and self-compassion Instagram images on women’s body image, self-compassion, and mood. Body Image 22, 87–96. doi: 10.1016/j.bodyim.2017.06.004 28689104

[ref50] Srivastava P. Michael M. L. Manasse S. M. Juarascio A. S. (2020). Do momentary changes in body dissatisfaction predict binge eating episodes? An ecological momentary assessment study. Eating Weight Disord. 26, 395–400. doi: 10.1007/s40519-020-00849-z, PMID: 31989487

[ref51] Stice E. (2016). Interactive and mediational etiologic models of eating disorder onset: evidence from prospective studies. Annu. Rev. Clin. Psychol. 12, 359–381. doi: 10.1146/annurev-clinpsy-021815-093317, PMID: 26651521

[ref52] Stice E. Shaw H. E. (2002). Role of body dissatisfaction in the onset and maintenance of eating pathology. J. Psychosom. Res. 53, 985–993. doi: 10.1016/s0022-3999(02)00488-9, PMID: 12445588

[ref53] Sumter S. R. Cingel D. Hollander L. (2022). Navigating a muscular and sexualized Instagram feed: an experimental study examining how Instagram affects both heterosexual and non-heterosexual men’s body image. Psychol. Pop. Media 11, 125–138. doi: 10.1037/ppm0000355

[ref54] Tatangelo G. McCabe M. Mellor D. Mealey A. (2016). A systematic review of body dissatisfaction and sociocultural messages related to the body among preschool children. Body Image 18, 86–95. doi: 10.1016/j.bodyim.2016.06.003, PMID: 27352102

[ref55] Thompson JK Heinberg LJ Altabe M Tantleff-Dunn S (1999). Exacting beauty: Theory, assessment, and treatment of body image disturbance. Washington, DC: American Psychological Association, doi: 10.1037/10312-000.

[ref56] Tiggemann M. (2002). “Media influences on body image development” in Body image: A handbook of theory, research, and clinical practice: A handbook of science, practice, and prevention. eds. Cash T. F. Pruzinsky T. (New York, NY: Guilford Publications), 91–98.

[ref57] Tiggemann M. Anderberg I. (2020). Muscles and bare chests on Instagram: the effect of influencers’ fashion and fitspiration images on men’s body image. Body Image 35, 237–244. doi: 10.1016/j.bodyim.2020.10.001, PMID: 33157398

[ref58] Tiggemann M. Zaccardo M. (2015). “Exercise to be fit, not skinny”: the effect of fitspiration imagery on women’s body image. Body Image 15, 61–67. doi: 10.1016/j.bodyim.2015.06.003, PMID: 26176993

[ref59] Tiggemann M. Zaccardo M. (2016). ‘Strong is the new skinny’: a content analysis of #fitspiration images on Instagram. J. Health Psychol. 23, 1003–1011. doi: 10.1177/1359105316639436, PMID: 27611630

[ref60] Tylka T. L. (2011). Refinement of the tripartite influence model for men: dual body image pathways to body change behaviors. Body Image 8, 199–207. doi: 10.1016/j.bodyim.2011.04.008, PMID: 21664886

[ref61] Vega A. V. Ramos L. M. Barrios M. L. Quintero M. V. (2015). Imagen del cuerpo en adultos mayores. (Body image in elderly adults). Salud En Movimiento 7, 4–10.

[ref62] Wade T. J. (2000). Evolutionary theory and self-perception: sex differences in body esteem predictors of self-perceived physical and sexual attractiveness and self-esteem. Int. J. Psychol. 35, 36–45. doi: 10.1080/002075900399501

[ref020] Watson A. Murnen S. K. College K. (2019). Gender differences in responses to thin, athletic, and hyper-muscular idealized bodies. Body Image, 30, 1–9. doi: 10.1016/j.bodyim.2019.03.010 31071678

